# Tracing Asian Seabass Individuals to Single Fish Farms Using Microsatellites

**DOI:** 10.1371/journal.pone.0052721

**Published:** 2012-12-20

**Authors:** Gen Hua Yue, Jun Hong Xia, Peng Liu, Feng Liu, Fei Sun, Grace Lin

**Affiliations:** Molecular Population Genetics Group, Temasek Life Sciences Laboratory, 1 Research Link, National University of Singapore, Singapore, Singapore; Swansea University, United Kingdom

## Abstract

Traceability through physical labels is well established, but it is not highly reliable as physical labels can be easily changed or lost. Application of DNA markers to the traceability of food plays an increasingly important role for consumer protection and confidence building. In this study, we tested the efficiency of 16 polymorphic microsatellites and their combinations for tracing 368 fish to four populations where they originated. Using the maximum likelihood and Bayesian methods, three most efficient microsatellites were required to assign over 95% of fish to the correct populations. Selection of markers based on the assignment score estimated with the software WHICHLOCI was most effective in choosing markers for individual assignment, followed by the selection based on the allele number of individual markers. By combining rapid DNA extraction, and high-throughput genotyping of selected microsatellites, it is possible to conduct routine genetic traceability with high accuracy in Asian seabass.

## Introduction

Food traceability is becoming increasingly important in ensuring food-safety in the agrifood industry [1], [2], [3], [4], [5]. As a number of traceability concepts and technologies are available [4], consideration needs to be given to the reliability and precision of traceability systems. Previous experience has shown that conventional tagging and labelling systems are prone to high error rates and may not have sufficient reliability and precision [1], [6]. DNA technology can overcome these existing problems in traditional labelling systems by tracking animals and their products through their DNA. This enables the tracking of any food products through the supply chain back to the source animals, and offers high reliability and precision of traceability [5], [7]. The precision and reliability of a DNA-based traceability system depend on the number and type of DNA markers used in the system [5]. DNA-based traceability requires collection of samples for extracting DNA, and specialized facilities to detect the DNA [2]. Although RAPD and AFLP markers have been used in genetic traceability of food products [8], [9], genotyping markers is usually tedious, and results of RAPD and AFLP analyses are not highly reproducible [10]. Microsatellites [11], which are short (2–6 bp) tandemly repetitive DNA sequences, are the markers of choice for traceability [2] because of their high abundance, high polymorphism and ease of scoring by PCR. Automatic genotyping of PCR products amplified with fluorescently labelled primers, and automated DNA sequencers considerably increases efficiency and precision of genotyping microsatellites and decreases the cost for genotyping [12]. Recently, single nucleotide polymorphism (SNPs) has been tested for genetic traceability [13]. A study showed that identification of highly informative SNP loci from large panels could provide a powerful approach to delineate genetic relationships at the individual and population levels [13]. However, for most aquaculture species, the number of SNPs is limited [14].

Aquaculture is the fastest increasing sector in agriculture. According to FAO's recent statistics [15], international trade of aquaculture fish products has reached a record high. Aquaculture plays a very important role in the economy of Asian countries [15]. The future and sustainable development of aquaculture will be progressively more market driven, and will rely heavily on its capacity to meet consumers' expectations. Therefore, the establishment of a comprehensive traceability system within the aquaculture industry is becoming increasingly important [1], [3]. Methods for tracing escapes to single fish farms using microsatellites were reported in salmon and rainbow trout [16], [17], [18], [19]. A simulation study showed that at least 15 microsatellites were required to reach 95% correct assignment decisions [20]. The number of markers required differs from species to species, and depends on the many factors such as genetic diversity and population structure [20], [21]. Therefore, it is essential to examine the power of DNA markers for genetic traceability for each species. It is known that the quality of farmed fish, which can vary greatly between farms, is mainly influenced by the quality of farming environment (e.g. water quality), feeds used, feeding regimes and the culture methods implemented [22], [23]. Thus, there is a growing need to develop highly reliable, rapid and cost-effective molecular tools for discriminating among farmed fish cultured in different systems and/or in different geographical locations [3], [7].

The Asian seabass (*Lates calcarifer*) is a highly valued aquaculture fish species. It has been farmed in Southeast Asia and Australia since 1980s [24], and recently some countries in Europe, such as Germany, France, as well as the USA, have started to culture this species [24]. In this species, a large number of DNA markers have been characterized [25], [26], [27], mapped to linkage maps [28], [29] and applied to study population structure [30], [31], to map QTL [28], [32] and to conduct parentage analysis for selective breeding [33]. However, no DNA markers have been used in genetic traceability in Asian seabass. The objective of this study is to develop a cost-effective and precise DNA-based tracking system for Asian seabass by evaluating the efficiency of each of the 16 selected microsatellites and their combinations, and by selecting the most powerful markers for individual assignment in four populations of Asian seabass. The selected microsatellites, in combination with the rapid and cost-effective method of DNA extraction developed previously [34], as well as the automatic genotyping of PCR products with DNA sequencers, will enable the routine genetic traceability with high accuracy in Asian seabass.

## Materials and Methods

### Fish samples

Fin clips of all Asian seabass breeding fishes (i.e. spawners) from three local farms (MAC, FARM-1 and FARM-2) were collected and stored in 75% ethanol. The MAC, FARM-1, and FARM-2 contained 104 (51 males and 53 females), 148 (66 males and 82 females) and 40 (20 males and 20 females) spawners respectively. The spawners of MAC were caught from the wild at sea near Singapore. For sampling spawners of Asian seabass, no specific permits were required for the described field studies. The spawners of FARM-1 originated from Indonesia, while the spawners of the FARM-2 were a mixture of different origins, including a few individuals from the western part of Australia. In addition, we obtained fin clip samples of 62 wild Asian seabass from the western part of Australia from 2003–2005. However, the exact sampling locations were not known. From the three local farms (MAC, FARM-1 and FARM-2), we collected the fin clips of 88, 96 and 96 juveniles (three months post hatch) respectively, and stored them in 75% ethanol. We also obtained 88 fin clips from offspring from a hatchery located in Darwin, Australia in 2006, and stored them in 75% ethanol. The spawners in the hatchery located in Darwin originated from the wild of the western part of Australia.

### DNA extraction and genotyping of microsatellites

DNA of each sample was isolated on 96-well plates using a very rapid and cost effective method developed in our laboratory previously [34].

Sixteen microsatellites (Lca002, Lca016, Lca020, Lca021, Lca040, Lca050 Lca057, Lca058, Lca062, Lca063, Lca064, Lca069, Lca070, Lca074, Lca086 and Lca098) were selected from a set of 27 markers that were characterized previously [35]. One primer of each pair was labelled with a fluorescent dye (either Fam or Hex or Ned) at the 5′ end of the primer. The PCR reaction for each sample consisted of 10 ng of genomic DNA, 0.5 units of *Taq* polymerase (Finnzymes, Vantaa, Finland), 1x PCR buffer containing 1.5 mM MgCl_2_, 0.2 µM dNTPs, and 50 nM of each primer. PCR was conducted on PTC-100 PCR machines (MJ Research, CA, USA) under the following conditions: 2 min denaturation at 94°C; 35 cycles of 30 s at 94°C, 30 s at 55°C and 30 s at 72°C and a final extension at 72°C for 10 min. PCR products were analyzed on an ABI3730xl DNA sequencer (Applied Biosystems, Foster City, USA). Fragment sizes were analyzed against the ROX-500 standard using GeneMapper 4.1 (Applied Biosystems, Foster City, USA). Genotypes were exported to Excel table for later data analysis.

### Statistical analysis

Allele number (A), expected (He) and observed (Ho) heterozygosity, and fixation index (f) of each microsatellite were estimated using the software Genetic Data Analysis (GDA) [36]. Allelic richness, a parameter for allelic diversity independent of sample size, was estimated with the software FSTAT [37] for each population. Polymorphism information content (PIC) and the exclusion probabilities (NE-1) of each microsatellite were calculated with the software CERVUS 3.0 [38]. *F*_ST_ between populations, statistical significance of population pairwise *F*_ST_ and molecular variance (AMOVA) were analyzed using the software ARLEQUIN 3.1 [39]. In AMOVA, the total variance is partitioned into separate components, each of which describes the proportion of the total variance at distinct hierarchical levels (within population and among populations). Ratios of the variance components can then be used to define population structure. Significance was tested by comparing observed values to null distribution generated by permutation using 10,000 replicates.

Two software WHICHRUN [40] and GENECLASS [41] were used for individual assignment. WHICHRUN performed individual assignment to populations based on a maximum likelihood method. The GENECLASS software [41] can implement three individual assignment tests: Bayesian-based approach, assignment based on reference population allele frequencies and assignment based on genetic distance. The base line used for the assignment in GENECLASS was the spawners from the four populations. As the Bayesian-based approach is commonly used, we used this method for our current analysis.

The software WHICHLOCI [42] was used to examine the assignment score for each marker and their combinations, and to select the most powerful microsatellites for individual assignment. In order to examine the power of single markers for individual assignment, we tested different methods for selecting markers: (1) based on the allele number of single markers, (2) based on the expected heterozygosity, and (3) based on the assignment score estimated using the software WHICHLOCI. We combined 2–16 markers (from the marker with the highest to the lowest allele number, expected heterozygosity and assignment score, respectively) to test the power of combinations of single markers for assignment. The base populations were the spawners from the four populations, whereas the juveniles were the individuals to be assigned.

## Results

### Polymorphisms and assignment power of 16 microsatellites

Sixteen microsatellites were individually examined for their polymorphisms and power for individual assignment in four Asian seabass populations consisting of 354 spawners. The polymorphisms and power for individual assignment of the 16 markers are shown in Table 1. The allele number of these markers ranged from 6 for Lca069 to 26 for Lca086, with an average allele number of 13.3. The average observed heterozygosity was 0.70, while the expected heterozygosity was 0.78. At the locus Lca086, the majority (Ho  = 0.91) of the 354 individuals were heterozygous, whereas at the locus Lca050, only 47% (Ho  = 0.47) of the 354 spawners were heterozygous. The polymorphism information content (PIC) varied from 0.41 for Lca050 to 0.91 for Lca058. The fixation index of 16 markers averaged at 0.08, with a range from 0.00 for Lca070 to 0.32 for Lca063. The non-exclusion probability (NE-I) ranged from 0.34 for Lca050 to 0.01 for Lca086. Assignment scores of individual markers were estimated with the software WHICHLOCI. Lca016 showed the highest assignment score (10.8), while Lca050 displayed the lowest score (4.2) (Table 1).

**Table 1:** Genetic variation and assignment power of 16 microsatellite loci in the four studied populations of Asian seabass.

Locus	Allele no.	Ho	He	PIC	NE-I	Score*	f
LCA002	12	0.65	0.73	0.70	0.11	5.1	0.11
LCA016	14	0.69	0.83	0.82	0.04	10.8	0.18
LCA020	13	0.72	0.80	0.77	0.07	6.1	0.10
LCA021	7	0.87	0.83	0.80	0.05	5.0	−0.06
LCA040	9	0.63	0.78	0.75	0.08	8.1	0.20
LCA057	13	0.74	0.79	0.76	0.07	6.6	0.06
LCA058	22	0.82	0.91	0.90	0.02	6.9	0.09
LCA062	17	0.73	0.89	0.88	0.02	8.0	0.18
LCA063	9	0.49	0.73	0.69	0.12	8.5	0.32
LCA064	12	0.72	0.81	0.78	0.06	5.5	0.10
LCA069	6	0.70	0.78	0.74	0.08	6.6	0.09
LCA074	11	0.58	0.64	0.60	0.17	2.8	0.09
LCA086	26	0.91	0.91	0.91	0.01	7.7	0.01
LCA098	19	0.69	0.77	0.75	0.07	7.8	0.10
Lca070	10	0.76	0.76	0.72	0.10	4.5	0.00
Lca050	13	0.47	0.45	0.41	0.342	4.2	0.03
Mean	13.3	0.70	0.78	0.77	1.2E- 19	-	0.08

Ho: observed heterozygosity; He: expected heterozygosity; PIC: polymorphism information content; NE-1: Non-exclusion probability (identity); Score*: Assignment score calculated using the software WHICHLOCI [42] and f: Fixation index.

### Genetic diversity and relationships among the four populations

The mean number of alleles, observed and expected heterozygosity, and fixation index of 16 microsatellites in each of the four populations are presented in Table 2. The Australian population and FARM-1 showed more alleles than the other two local farms in Singapore.

**Table 2:** Number of samples (N), mean number of alleles (A), mean allelic richness (Ar), observed (Ho) and expected (He) heterozygosity, and fixation index (f) estimated with 16 microsatellites in four populations of Asian seabass.

Population	N	A	Ar	Ho	He	f
AUS	62	9.75	8.91	0.60	0.74	0.18
MAC	104	8.50	7.54	0.72	0.73	0.01
FARM-1	148	9.75	7.78	0.70	0.72	0.03
FARM-2	40	7.19	7.19	0.72	0.74	0.03

*F_ST_* analysis showed that the Australian population was significantly (*F_ST_*  = 0.11–0.13, *P*<0.01) different from the three populations in Singapore. The differentiation of the three local populations was small (*F_ST_*  = 0.048–0.067), but statistically significant (*P*<0.05). AMOVA also revealed that the Australia population was significantly different from the three local farms (among population variation  = 8.37%, *P*<0.01).

### Methods of selecting markers and the number of markers required for assigning individuals to single propulsions

We examined the power of marker combinations for individual assignment by selecting markers based on three parameters (i.e. assignment score, allele number and expected heterozygosity). Using both ML and BS methods, we had the lowest correct assignment of the juveniles from FARM-1. Therefore, in the following results, we showed the percentage of correct assignment for all juveniles (Overall) and juveniles from FARM-1.

**Figure 1:**
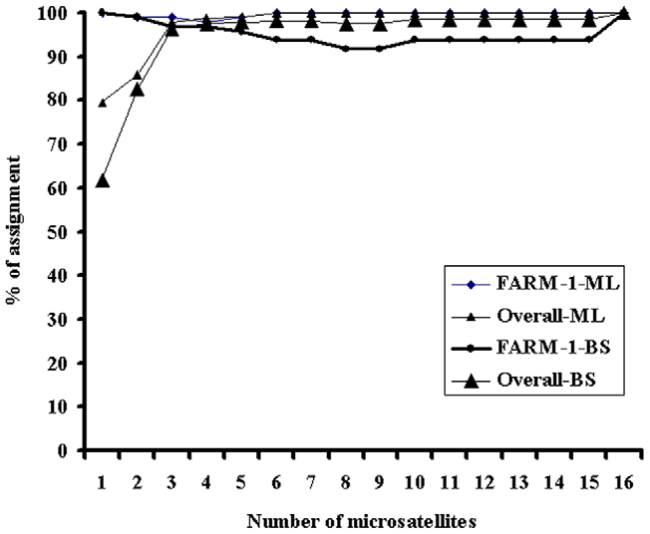
The correct assignment percentage of individuals corresponding to the number of loci selected on the basis of the ranking of score (from high to low) obtained by WHICHLOCI. The marker order was Lca016, Lca063, Lca040, Lca062, Lca098, Lca086, Lca058, Lca057, Lca069, Lca020, Lca064, Lca002, Lca021, Lca070, Lca050 and Lca074. The individual assignment was performed using the Bayesian (BS) and maximum likelihood (ML) methods.

Using only three most powerful markers (Lca016, Lca062 and Lca021) selected based on their assignment scores, 97.8% and 96.7% of juveniles could be correctly assigned to their original populations using the ML and BS methods, respectively (Figure 1). With all the 16 markers, all 368 juveniles could be correctly assigned to their original populations.

**Figure 2:**
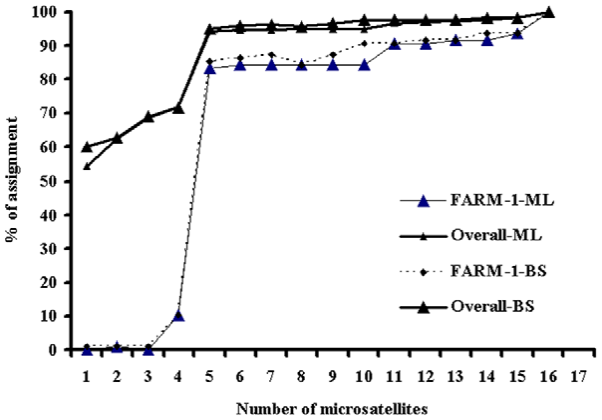
The percentage of correct assignment of individuals corresponding to the number of loci selected on the basis of the ranking of allele number of the markers from high to low. The marker order was Lca086, Lca058, Lca098, Lca062, Lca016, Lca050, Lca057, Lca020, Lca064, Lca002, Lca074, Lca070, Lca063, Lca040, Lca021 and Lca069. The assignment was conducted using the Bayesian (BS) and maximum likelihood (ML) methods.

Based on the allele number of individual markers, using five most polymorphic markers (Lca086, Lca098, Lca058, Lca062 and Lca016) together with the ML and BS methods, the percentage of overall correct assignments reached over 95% (Figure 2). Most of the individuals which could not be correctly assigned were from FARM-1.

**Figure 3:**
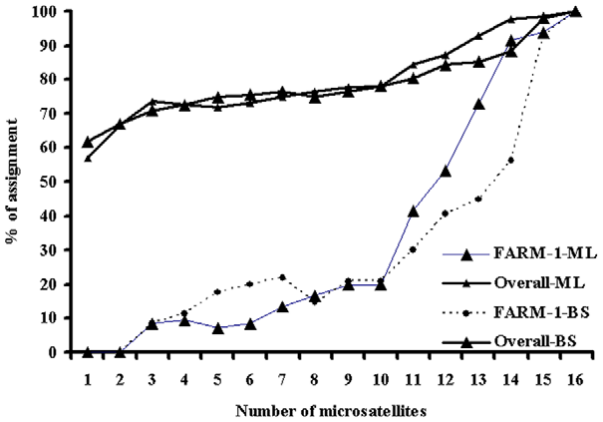
The percentage of correct assignment of individuals corresponding to the number of loci selected on the basis of expected heterozygosity (from high to low expected heterozygosity). The marker order was Lca086, Lca058, Lca062, Lca021, Lca016, Lca064, Lca057, Lca020, Lca069, Lca040, Lca098, Lca070, Lca063, Lca074, Lca002 and Lca050. The individual assignment was carried out using the Bayesian (BS) and maximum likelihood (ML) methods.

Based on the expected heterozygosity of individual markers, the percentage of correct assignment reached 95% by using up to 15 markers (Figure 3).

## Discussion

### Microsatellites as markers for genetic traceability in Asian seabass

Traceability is critically important in ensuring food safety and building consumers' confidence [7]. Although physical labels have been used for traceability for a very long time, they are still not very reliable, as physical labelling can be easily lost and changed [1]. Traceability using DNA markers is more reliable [4], and has been used in the livestock industry [2]. In the seafood industry, genetic traceability using DNA markers is just in its infancy [1], although genetic tracing had been used in aquaculture, such as tracking escapees of salmon and rainbow trout [16], [19]. In this study, we tested the power of 16 microsatellites and their combinations in assigning individuals to their original populations. In general, the 16 microsatellites were highly polymorphic in the four populations. Using only the three most efficient microsatellites selected based on the assignment score, over 95% of 368 individuals could be correctly assigned to their original populations. These data suggest that these 16 microsatellites could be used for future routine individual/product assignment of Asian seabass and that the selection of most efficient markers for parentage assignment is essential.

### Population structure influenced the power of DNA markers for traceability

The genetic differentiation between the Australian and Asian populations was much bigger than that among the three Asian populations, which is in agreement with the results of previous studies on genetic relationships of Asian seabass populations [30], [31]. It is known that population structure and relationships influenced the power of DNA markers for traceability [20]. In this study, the population FARM-1 was the most diverse, containing the majority of alleles. The juveniles originating from this FARM-1 were assigned to other populations when only a few microsatellites were used, suggesting that when selecting DNA markers for individual assignment, population structure and relationships must be taken into account. It is advisable to conduct some pilot studies to examine the efficiency of DNA individual markers to select the most efficient DNA markers for routine genetic traceability in pupations where genetic traceability to be conducted.

### Other factors influencing the power of DNA markers for traceability

Besides the structure of populations of spawners, other factors such as the allele number of markers, heterozygosity of markers, and assignment score of individual markers, number of markers [21], [43] and other factors (e.g. statistical methods) may also influence the efficiency of individual DNA markers for individual assignment,. In this study, the two statistical methods showed similar results. Therefore, we only analysed the effects of the allele number, heterozygosity and assignment score of individual markers on the efficiency of individual assignment. To reach over 95% of correct assignment, based on the assignment scores estimated with the software WHICHLOCI, only three markers with the highest score were required, whereas based on allele number and expected heterozygosity, at least 6 and 15 microsatellites were required, respectively. These data suggest that selection of markers based on the assignment score estimated with the software WHICHLOCI is most effective for individual assignment. Therefore, in practice, to accomplish high efficiency of assignment of individuals, it is essential to conduct some small-scale feasibility studies to examine the power of markers for individual assignment using the software WHICHLOCI.

## Conclusions

We have tested the efficiency of 16 microsatellites and their combinations in assigning individuals to populations of Asian seabass where they originated. Three most effective microsatellites were required to assign over 95% of fish to the correct populations. Selection of markers based on the assignment score estimated with the software WHICHLOCI was most effective in choosing markers for individual assignment, followed by the selection based on the allele number of individual markers. Therefore, for routine genetic traceability, it is essential to conduct some small-scale feasibility studies to select the most efficient DNA markers. By combining the rapid DNA extraction method developed previously by us [34] and automatic genotyping of selected microsatellites using sequencers, it is possible to conduct routine genetic traceability with high accuracy in Asian seabass.
